# Neuromechanical Properties of the Vastus Medialis and Vastus Lateralis in Adolescents With Patellofemoral Pain

**DOI:** 10.1177/23259671231155894

**Published:** 2023-06-07

**Authors:** Marion Crouzier, François Hug, Frances T. Sheehan, Natalie J. Collins, Kay Crossley, Kylie Tucker

**Affiliations:** * Laboratory “Movement, Interactions, Performance” (UR 4334), University of Nantes, Nantes, France.; † Université Côte d’Azur, LAHMESS, Nice, France.; ‡ Rehabilitation Medicine Department, National Institutes of Health Clinical Center, Bethesda, Maryland, USA.; § School of Health and Rehabilitation Sciences: Physiotherapy, Faculty of Health and Behavioural Sciences, The University of Queensland, St. Lucia, Queensland, Australia.; ∥ La Trobe Sport and Exercise Medicine Research Centre, La Trobe University Melbourne, Australia.; ¶ School of Biomedical Sciences, Faculty of Medicine, The University of Queensland, St. Lucia, Queensland, Australia.; *Investigation performed at School of Biomedical Sciences, Faculty of Medicine, University of Queensland, St. Lucia, Australia.*

**Keywords:** electromyography, moment arm, muscle coordination, musculoskeletal disorder, physiological cross-sectional area

## Abstract

**Background:**

An alteration in the force distribution among quadriceps heads is one possible underlying mechanism of patellofemoral pain. However, this hypothesis cannot be directly tested as there are currently no noninvasive experimental techniques to measure individual muscle force or torque in vivo in humans. In this study, the authors considered a combination of biomechanical and muscle activation measures, which enabled us to estimate the mechanical impact of the vastus medialis (VM) and vastus lateralis (VL) on the patella.

**Purpose/Hypothesis:**

The purpose of this study was to determine whether the relative index of torque distribution for the VM and VL differs between adolescents with and without patellofemoral pain. It was hypothesized that, relative to the VL, the VM would contribute less to knee extension torque in adolescents with patellofemoral pain compared with controls.

**Study Design:**

Cross-sectional study; Level of evidence, 3.

**Methods:**

Twenty adolescents with patellofemoral pain and 20 matched control participants were included (38 female; age, 15.3 ± 1.8 years; weight, 58 ± 13 kg; height, 164 ± 8 cm). Muscle volumes and resting moment arms were quantified from magnetic resonance images, and fascicle lengths were obtained from panoramic B-mode ultrasonography. Muscle activation was estimated using surface electromyography during submaximal isometric tasks (wall-squat and seated tasks). Muscle torque was estimated as the product of muscle physiological cross-sectional area (ie, muscle volume/fascicle length), muscle activation (normalized to maximal activation), and moment arm.

**Results:**

Across tasks and force levels, the relative contribution of the VM to the overall medial and lateral vastii torque was 31.0% ± 8.6% for controls and 31.5 ± 7.6% for adolescents with patellofemoral pain (group effect, *P* > .34).

**Conclusion:**

For the tasks and positions investigated in this study, the authors found no evidence of lower VM torque generation (relative to the VL) in adolescents with patellofemoral pain compared with controls.

Patellofemoral pain is a musculoskeletal condition presenting as pain around or behind the patella that is aggravated by patellofemoral joint-loading activities such as deep knee flexion or repetitive flexion/extension.^10^ Pathological patellofemoral kinematics (patellar maltracking) is widely accepted as an underlying mechanism of patellofemoral pain.^14^ Altered force distribution between the medial (vastus medialis [VM]) and the lateral (vastus lateralis [VL]) heads of the quadriceps may contribute to maltracking and patellofemoral pain.^19^ The effect of altered quadriceps force distribution on patellar kinematics has been demonstrated in cadaveric^37^ and animal^16,17^ experiments. There are no experimental techniques to noninvasively measure individual muscle force in vivo for either humans or animals. However, experiments that employ a VM nerve motor branch block^35^ or selective VM or VL muscle electrical stimulation^24^ support the relationship between altered force distribution and patellar maltracking.

Most previous studies that aimed to evaluate the muscle force balance between VM and VL in patients with patellofemoral pain have used indirect approaches (eg, electromyography [EMG]) and have not reached consensus in their findings. Several studies reported an imbalance of activation amplitude biased toward VL in patellofemoral pain,^29,34^ but just as many studies reported no imbalance of activation.^8,23,31^ The problem with the isolated EMG approach is that it only measures muscle activation, which cannot account for other factors that contribute to muscle force and mechanical impact on the patella, such as physiological cross-sectional area (PCSA)^18^ and moment arms.^41^

A better understanding of the mechanisms that contribute to onset and persistence of pain is necessary to improve our ability to successfully devise and apply rehabilitation strategies.^36^ In the current study, we aimed to measure a series of parameters that contribute to the torque applied to the patella during isometric contractions, specifically VM and VL activation level, PCSA, and moment arm (relative to the patella). We hypothesized that the combination of all such parameters would highlight a lower VM contribution to knee extension torque relative to VL in people with patellofemoral pain compared with controls. The study was conducted on an adolescent population, as <5% of published studies on patellofemoral pain are devoted to adolescents^32^ despite the high prevalence of patellofemoral pain in this population.

## Methods

### Participants

Participants were recruited from the community via social media and social networks, newsletters from sporting groups/schools, and posters at sporting events. Eligibility was first determined by a physical therapist (M.C.) via telephone/email and then confirmed during a physical screening. Informed consent was obtained from the participant and guardian after eligibility was confirmed and before any testing occurred. Ethics committee approval was received for the study protocol.

Inclusion criteria for all participants were (1) age between 12 and 18 years, (2) physically able to undertake testing procedures, and (3) no contraindications to magnetic resonance imaging (MRI). In addition, criteria for adolescents with patellofemoral pain inclusion were (1) anterior knee pain of nontraumatic origin that was rated for maximum pain experienced in the preceding week as ≥2 on an 11-point numeric rating scale (from 0 = no pain to 10 = maximal pain) and was aggravated by joint-loading activities (eg, squatting, stair climbing), and (2) pain duration ≥2 months. Patellofemoral pain exclusion criteria were (1) concomitant pain at sites other than the anterior knee; (2) history of knee, hip or spine surgery, or other suspected knee joint pathology (eg, Osgood-Schlatter disease, arthritis, history of dislocation, patellar tendinopathy); (3) recent (≤1 year) intra-articular knee injections; and (4) planned lower limb surgery (eg, arthroscopy). Targeted recruitment of controls ensured that participants in each group were paired for age, sex, and body mass index (all within ±15%). All participants completed the Knee injury and Osteoarthritis Outcome Score for Children (KOOS–Child), and physical activity levels were matched to frequency per week and type of sport. The control group had no history of lower limb pain/injury for which they pursued treatment or required time off from work/sport/school and no pain on the day(s) of testing.

### Experimental Protocol

In addition to the clinical assessment performed by the physical therapist to determine eligibility, participants attended 2 sessions: (1) an MRI session during which images were obtained to measure muscle volumes and moment arms and (2) a laboratory session during which muscle activation (from surface EMG) and muscle fascicle length (from ultrasound images) data were obtained. All testing occurred within 25 days of the clinical assessment (2.5 ± 5.7 days).

### Magnetic Resonance Imaging

Volumetric acquisitions of the thighs were performed using 3-T MRI (Magnetom Prisma; Siemens Healthcare). Participants were supine with a cushion supporting their knee at 60° of flexion. Their feet were secured comfortably with a strap to maintain a consistent foot and lower limb alignment. A 3-dimensional (3D) steady-state Vibe sequence without fat suppression was used (repetition time, 9 ms; echo time, 2.26 ms; field of view, 338 × 450 mm^2^; voxel size, 0.88 × 0.88 × 2 mm; flip angle, 10 slices; thickness, 2 mm). This sequence enhanced our ability to identify separation between muscles, improving segmentation accuracy. The image volume ranged from the iliac crests to the articular surface of the lower border of the patella, using 2 slightly overlapping acquisitions. All data were saved in Digital Imaging and Communications in Medicine format.

#### Muscle Volume

**Figure 1.**
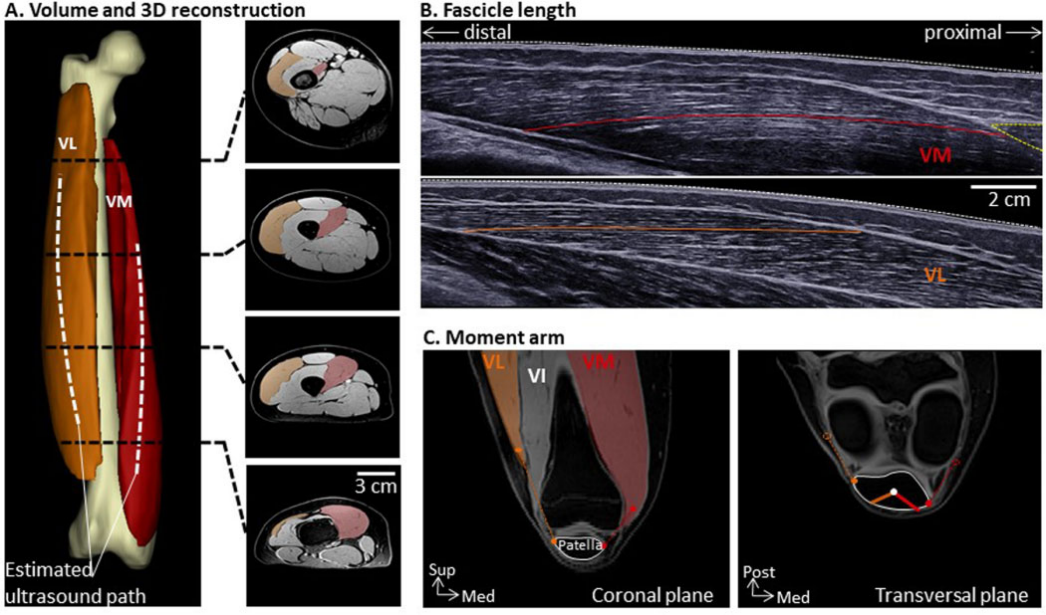
Experimental measures of physiological cross-sectional area (PCSA) and moment arm for vastus medialis (VM) and vastus lateralis (VL). (A) VM, VL, and femur 3-dimensional (3D) reconstruction from the magnetic resonance imaging acquisitions. VM and VL were manually segmented from each axial image. Their volumes were reconstructed using 3D image analysis software. Example of cross-sectional images are taken from 20%, 40%, 60%, and 80% of femoral length. (B) Fascicle length was determined from panoramic ultrasound images of VM and VL using a custom-made MATLAB script. Examples of 1 fascicle from each muscle are delineated. The yellow dotted line on the VM image indicates the sartorius, which was the anatomical landmark used to determine the probe’s line of travel on the skin for VM. The PCSA of each muscle was calculated as its volume divided by fascicle length. (C) VM and VL moment arms were defined as the perpendicular distance from the line of action of their respective tendon to the patellar centroid. Lines of action are shown in dashed lines. Two-dimensional projections of the VM and VL moment arms are shown by thick lines on the transverse view. Med, medial; Post, posterior; Sup, superior; VI, vastus intermedius.

MRIs were analyzed using 3D image analysis software (Mimics; Materialise NV). Both VM and VL were segmented manually in every slice between the muscle origin and insertion (Figure 1A). Previous work from cadaveric studies reported that the vastus intermedius (VI) and VL are fused in the more proximal regions.^39^ As proposed by Barnouin et al,^5^ we maintained a *bean* shape for VL, consistently using the visible landmarks on the preceding and subsequent images to assist the segmentation when the boundary between the VL and VI was indistinct. The volume of each muscle was calculated from 3D reconstruction using Mimics software.

#### Muscle Moment Arms

The moment arm magnitude was defined as the perpendicular distance from the line of action of each VM and VL tendon to the patellar centroid. VM and VL tendon lines of action were defined as the unit vector from the tendon’s patellar insertion to its muscular origin. The tendon insertion was defined as the medial (VM tendon) or lateral (VL tendon) pole of the patella (Figure 1C). To minimize the influence of a single slice, the origin of the tendon was defined as the average geometric center of the distal-most 10 images of VM muscle (VM tendon) or VL muscle (VL tendon). Coordinates for each point of interest (patellar centroid, VM and VL tendon insertions and origins) were extracted from MRI using *Medical Image Processing, Analysis and Visualization*^26^ and were imported to a custom-made MATLAB program (Version 9.8; The MathWorks Inc) for moment arm calculation.

### Laboratory Session

#### Knee Extensor Torque

**Figure 2.**
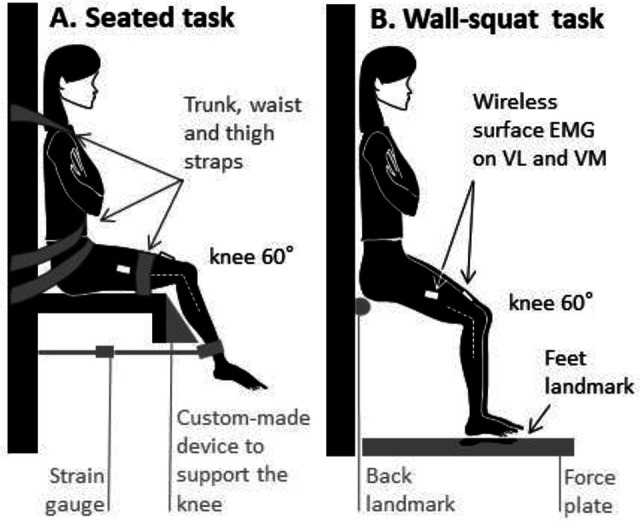
Position for the (A) seated and (B) wall-squat isometric knee extension tasks. Muscle activity was measured using surface electromyography (EMG) electrodes placed on the vastus medialis (VM) and vastus lateralis (VL). A knee angle of 60° was maintained in both tasks. Extension force was measured with a strain gauge in panel A, and the center of pressure was determined using force plates in panel B. For the wall-squat task, once the position was determined, placement positions of the back and feet were marked on the wall and force plate, respectively, to facilitate returning to the same positions between contractions.

Participants first sat on a supportive chair, with hips at 90° and knees at 60° (when both joints are at 0°, the lower limb is straight and aligned with the torso) (Figure 2A). This angle was chosen because there is evidence of no force deficit in adolescents with patellofemoral pain at this angle during maximal voluntary isometric knee extensions (maximal voluntary contractions [MVCs])^33^ and because no pain increase was observed during isometric contractions at this angle during pilot trials. Wide, supportive straps were firmly secured around the participant’s waist, thigh, and trunk to minimize contribution to knee extension from muscles other than the quadriceps. Knee extension force was measured with a strain gauge (LSB350; Futek) attached in series from the chair to the participant’s leg using a noncompliant strap at a known distance (25 ± 3 cm) from the apex of the patella. This distance was used to convert the measured force into a measure of knee extension torque. Force signals were amplified, sampled at 2000 samples per second (Power1401 Data Acquisition System; Cambridge Electronic Design), and displayed as force feedback for participants.

The participants performed a series of standardized warm-up tasks to familiarize themselves with the equipment and visual feedback of force. When ready, they performed 3 MVCs. For each MVC, participants increased their force from rest to maximum over 3 seconds and then held this contraction for ∼2 seconds before being instructed to rest for 120 seconds. A maximum of 10% difference in force between the 2 highest MVC trials was tolerated. If this was not achieved, additional MVCs were performed.

For the analyses, force was low-pass filtered at 10 Hz and MVC force was calculated as the maximal force over a 300-ms time window. The largest force obtained during these MVCs was then used to calculate the submaximal contraction targets for each participant. While seated in the same position, participants performed a series of submaximal isometric knee extensions to a target torque of 10%, 20%, and 40% of their MVC. Four trials of 8 seconds each were performed at each intensity in a randomized order, for a total of 12 submaximal contractions. Feedback of the target and actual force was provided using visual feedback displayed on a monitor in front of the participant.

Upon completion of the seated tasks, the participants moved to an upright wall-squat position, with hips at 120°, knees at 60°, and feet hip-width apart (Figure 2B). They stood on 2 force plates (Bertec), which were used to provide feedback regarding the center of pressure relative to their foot position on their test side. While in this wall-squat position, participants were asked to perform 2 tasks: move their center of pressure anterior (ie, more under their toes) or posterior (ie, more under their heels) without lifting any aspect of their feet from the force plate.

After performing a series of training tasks to familiarize themselves, four 6-second trials of each task (8 in total) were conducted in a counterbalanced order. As this task was fatiguing, a rest break of ≥30 seconds was provided after every 2 trials. During this break, the participants stood comfortably or could move around. After each MVC and seated and wall-squat task, participants rated the maximum knee pain that they experienced during the task on a visual analog scale (0 = no pain to 10 = maximal pain). None of the participants asked to stop the study because of pain.

#### Muscle Activation

For both the seated and the wall-squat tasks, myoelectrical activity was recorded with surface EMG electrodes (Trigno Flex; Delsys) placed over the VM and VL. Rectus femoris myoelectrical activity was also recorded, but it is not reported in this article. Before electrode application, the skin was cleaned with abrasive gel (Nuprep; D.O. Weaver & Co) and alcohol. The electrode was placed within mediolateral muscle boundaries at ∼80% (VM) and ∼66% (VL) of the muscle length distally. B-mode ultrasonography (Aixplorer; Supersonic Imagine) was used to facilitate the placement of the electrodes within muscle boundaries and longitudinally with respect to fascicle alignment. EMG data were amplified 1000 times, band-pass filtered between 10 and 499 Hz, and digitized at a sampling rate of 2079 samples per second using a Power1401 Data Acquisition System with Spike2 software (Cambridge Electronic Design). Raw EMG signals were first inspected for electrical noise and movement artifact.

For the seated task, 21 of the 480 trials (4 repetitions × 3 intensities × 40 participants) were excluded because of artifacts, leading to a mean of 3.8 ± 0.4 repetitions per participant and per level of contraction. For the wall-squat task, no data were excluded for EMG quality; however, data from 2 participants in the control group were excluded because of technical problems with the force-plate data. Data from their matched participant were withdrawn from the patellofemoral pain group in the reported analysis (18 participants remained in each group).

For each MVC trial, the root mean square (RMS) EMG was calculated over a moving 300-ms window with 99% overlap. The highest value among all trials was considered the maximal RMS EMG value (RMS EMG_max_). For the seated and wall-squat tasks, the RMS EMG was calculated over 4 seconds at the middle of the force plateau. All EMG data from submaximal contractions were normalized to RMS EMG_max_.

#### Muscle Fascicle Length

An ultrasound scanner coupled with a 50-mm linear probe (4-15 MHz; Aixplorer; Supersonic Imagine) was used in panoramic mode to record the images from which we estimated the VM and VL muscle fascicle lengths (Figure 1B), as previously described.^28^ Participants were at rest in the seated position (hip, 90°; knee, 80°) for this measure. This knee angle was chosen as it is the angle at which maximum knee torque can be produced.^25^ For VL, the ultrasound probe was placed at the middle of the muscle belly, and for VM, the probe was placed midway between the distal insertion (on the medial patellar border) and the proximal area where the VM becomes deep (between the rectus femoris and sartorius muscles). From these probe positions, the fascicle plane was identified as the direction for which the longest continuous fascicles were visible. A line was drawn on the skin from the distal to the proximal insertion along the fascicle path to assist real-time ultrasound imaging.

For both muscles, images were recorded as the probe moved along the drawn lines with minimal pressure applied to the skin. At least 2 high-quality images were recorded for each muscle. Analysis of the ultrasound images, during which the investigator (M.C.) was blinded to group assignment, was conducted in MATLAB. As some fascicles exhibited a small curvature, we used segmented lines to denote the fascicle (Figure 1B). The fascicle length was considered the cumulative distance between the 2 aponeuroses. We attempted to obtain 3 fascicles for each image, and a mean fascicle length was determined. From a total of 240 possible fascicle length measurements for each muscle (2 images × 3 fascicles × 40 participants), image quality was insufficient to obtain the fascicle length for 36 VM and 5 VL images. A mean of 5.1 ± 1.0 VM fascicles and a mean of 5.9 ± 0.5 VL fascicles were included per participant for analysis. A mean fascicle length was determined for each muscle.

### Patellar Torque Index

We considered the mechanical effect of VM and VL on the patella by quantifying a torque index:


Torque index = PCSA × normalized RMS EMG × moment arm,

where the torque index is in arbitrary units, PCSA is in square centimeters, normalized RMS EMG is in percentage (of RMS EMG_max_), and moment arm is in centimeters. The torque produced by the muscle was obtained by multiplying the torque index by the muscle-specific tension. Since it was not feasible to measure the specific tension, we assumed it was a constant across muscles.^30^ To interpret the distribution of torque between VM and VL, we calculated the VM/(VM+VL) ratio from our torque indices.

### Statistical Analysis

Skewness and kurtosis tests were used to test for a normal distribution (Stata Version 12.0; StataCorp LP). If distributions did not pass the normality test, data were transformed. Specifically, muscle torque indices were transformed using a log function. All data are reported as mean ± SD.

Statistical analyses were performed in Statistica (Version 7.0; Statsoft). Demographic data, results from KOOS–Child questionnaire, maximal knee extension torque, and pain/physical activity questionnaire scores were compared between groups using unpaired *t* tests. To determine whether adolescents with patellofemoral pain exhibited different torque indices relative to controls, we used a 3-way analysis of variance (ANOVA) (between-partipicant factor: group; within-partipicant factors: muscle and intensity for seated task, muscle and condition for the wall-squat task). For each task, the ratios of torque indices between the 2 muscles VM/(VM+VL) were compared between groups with a 2-way ANOVA (within-partipicant factor: intensity for the seated tasks and condition for the wall-squat task). When appropriate, post hoc analyses were performed using the Tukey honestly significant difference test. The level of significance was set at *P* ≤ .05.

## Results

A total of 42 adolescents met our inclusion criteria and volunteered to participate. Of these, 2 participants withdrew before completing the MRI part of the study. Data from the remaining 40 participants (20 adolescents with patellofemoral pain and 20 matched controls) are reported in Table 1. Participants were successfully matched between groups for sex, age, body mass index, and physical activity (*P* > .13 for all) (Table 1). The patellofemoral pain group reported significantly more pain and disability associated with their patellofemoral pain, as assessed by the KOOS–Child. Participants with patellofemoral pain scored significantly lower than controls on all subscales, with the function in sport and play ([57.8 ± 20.9]/100) and quality of life ([55.6 ± 18.8]/100) subscales demonstrating the worst scores.

**Table 1:** Demographic and Knee Pain–Related Characteristics*^a^*

	Control (n = 20)	PF Pain (n = 20)	*P*
Age, y	15.4 ± 2.0	15.3 ± 1.8	.87
Sex, female/male	19/1	19/1	N/A
Height, cm	162.9 ± 8.9	165.4 ± 6.7	.32
Body mass, kg	55.3 ± 10.8	61.2 ± 13.2	.13
Body mass index	20.7 ± 3.2	22.2 ± 3.7	.19
Physical activity, MET, min/d	45.1 ± 8.9	46.7 ± 6.7	.52
KOOS–Child (100 = healthy)			
Pain	96.9 ± 6.4	62.8 ± 16.0	**<.01**
Symptoms	95.2 ± 7.4	78.4 ± 11.6	**<.01**
Difficulty during daily activities	98.8 ± 2.9	80.7 ± 14.0	**<.01**
Function in sport and play	95.5 ± 8.6	57.8 ± 20.9	**<.01**
How has your injury affected your life?	NA	55.6 ± 18.8	N/A
Side used for measures, left/right	6/14	7/13	N/A
Side used to kick a ball, left/right	5/15	4/16	N/A

*^a^* Data are reported as mean ± SD or absolute values. Boldface *P* values indicate a statistically significant difference between groups (*P* ≤ .05). Participants were pair matched by sex, age, and body mass index, and then type and volume of physical activity. KOOS–Child, Knee injury and Osteoarthritis Outcome Score for Children; MET, metabolic equivalent of task; NA, not applicable; PF, patellofemoral.

The maximum torque produced during MVCs was similar between the control and patellofemoral pain groups (120 ± 41 vs 116 ± 29 N·m; *P* = .70). Mild, local knee pain was reported during MVCs by 2 participants in the control group (pain = [0.6 ± 0.3]/10) and 7 in the group with patellofemoral pain (pain = [2.6 ± 2.3]/10). During the seated tasks, pain was reported by 1 participant in the control group (pain = [0.2 ± 0.1]/10) and 4 participants in the group with patellofemoral pain (pain = 0.6 ± 0.3/10). During the wall-squat tasks, pain was reported by 2 participants in the control group (pain = 0.2 ± 0.1/10) and 8 participants in the group with patellofemoral pain (pain = 1.1 ± 0.9/10).

**Figure 3.**
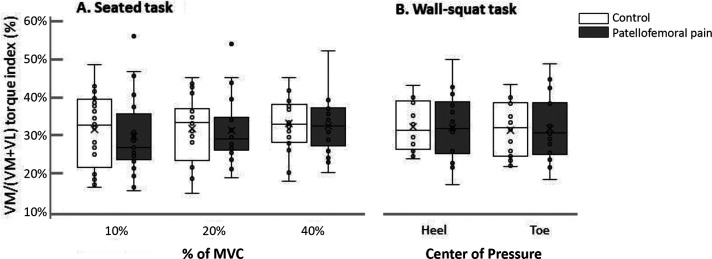
Distribution of torque between the vastus medialis (VM) and vastus lateralis (VL) (ie, VM/[VM+VL] ratio) measured during (A) seated tasks and (B) wall-squat tasks. Box plots show the minimum and maximum values (whiskers), first and third quartiles (top and bottom of the box), and medians (centerline). The X’s indicate mean values. MVC, maximal voluntary contraction.

When considering individual muscle torque indices, our results did not support our hypothesis that VM/(VM+VL) torque distribution would differ in adolescents with patellofemoral pain compared with controls (Table 2, Figure 3). There was no significant main effect of group (seated, *P* = .36; wall squat, *P* = .69), no group × muscle interaction (seated, *P* = .74; wall squat, *P* = .98), no group × intensity × muscle interaction (seated, *P* = .63), and no group × condition × muscle interaction (wall squat, *P* = .52). The VM/(VM+VL) torque index ratio confirmed the absence of between-group differences (seated, *P* = .72; wall squat, *P* = .95); there was no group × intensity interaction (seated, *P* = .78) and no group × condition interaction (wall squat, *P* = .65). Importantly, the VM torque index was lower than the VL torque index (all VM/[VM+VL] torque ratios, <32.6%), regardless of the task or group.

**Table 2:** Torque Indices (in Arbitrary Units) for the VM and VL in the Control and Patellofemoral Pain Groups*^a^*

	Control Group		Patellofemoral Pain Group	
	VL	VM	VL	VM
Seated task				
10% of MVC	837 ± 261	386 ± 169	725 ± 277	311 ± 194
20% of MVC	1311 ± 463	596 ± 232	1239 ± 459	569 ± 292
40% of MVC	2305 ± 846	1103 ± 391	2258 ± 782	1077 ± 443
Wall-squat task				
Heel	2765 ± 2021	1268 ± 1024	2220 ± 1023	1016 ± 458
Toe	2000 ± 1671	839 ± 712	1522 ± 799	685 ± 327

*^a^* Data are reported as mean ± SD. MVC, maximal voluntary contraction; VL, vastus lateralis; VM, vastus medialis.

## Discussion

To our knowledge, this is the first study to estimate VM and VL torques in adolescents with patellofemoral pain. The novelty of this work is 2-fold: (1) our focus on adolescent-onset patellofemoral pain and (2) our primary measures for torque indices that more comprehensively represent the muscles’ ability to generate a force on the patella. Unlike previous indirect approaches that have inferred muscle force from EMG alone, our indices account for multiple key parameters affecting torque generation (muscle activation, PCSA, and moment arms). This approach enables us to demonstrate that VL produces more torque than VM during isometric contractions in adolescents and that the amplitude of the imbalance in muscle forces does not differ between those with and without patellofemoral pain.

Our torque indices were not significantly different between adolescents with patellofemoral pain and controls. Of note, considering each parameter individually (muscle activation, PCSA, and moment arms) also revealed no differences between groups. Importantly, by considering the mean amplitude of muscle activation (EMG), we did not account for small variations in VM and VL activation that exist during a sustained contraction. There is some evidence that VM and VL motor unit synchronization^26^ differs in people with knee pain, and it is suggested that strength of covariation of activation of VM and VL is related to joint stress.^1^ Data collected on animals demonstrate that in different conditions, such as VL denervation^2^ or artificial lateral patellar pull from a surgically inserted spring,^6^ the covariation between VM and VL activation is systematically stronger than that with the VI or rectus femoris. As VM and VL coactivation minimizes the net mediolateral patellar force, it supports the notion that the central nervous system prioritizes joint integrity (ie, avoids excessive joint stress) to the detriment of task performance.^1^ Further studies need to explore whether the strength of covariation between VM and VL activation is lower in adolescents with patellofemoral pain than controls and how this relates to internal stresses within the patellofemoral joint. The VM and VL PCSA and moment arms also did not differ between groups, which match findings in adults.^4,13,42^

The absence of differences between groups cannot discount that other mechanical factors may contribute to adolescent-onset patellofemoral pain, since other biomechanical parameters may contribute significantly to healthy patellar tracking (reviewed previously^3^). For example, it is possible that joint geometry (eg, patella alta, trochlear groove depth^38^) or tendon properties are different between groups. The latter is supported by evidence that VM tendon strain is lower in adults with patellofemoral pain compared with controls (while VL tendon strain is similar) when assessed during similar tasks and a similar position as in the current study.^40^ If such an observation is because of a change in stiffness of the VM tendon and not in force of the VM muscle, the specific VM tendon property changes would affect patellar tracking for a given muscle force distribution. Also, other soft tissues should be considered. For example, the VI tendon portion builds a strong counterpart to the medial-acting forces on the patella because of an aponeurotic connection with the VM.^15^

It is important to recognize that the current findings may not be generalizable to the adult population for understanding the disease and its clinical implications. In adolescents, pain is initiated during a time of rapid bone growth and neuromuscular adaption. The length of disease is far shorter in adolescents, and there are differences in how pain presents in adults.^32^ For example, patellofemoral shape changes observed in adolescents are not commonly found in adults,^12^ and the spectrum of patellar maltracking in adolescent patellofemoral pain is also different than in adults.^9,21^ In addition, our study identified no difference in knee extension peak torque between groups, while “quadriceps weakness” has been previously described as a characteristic of adult patellofemoral pain.^22^ Greater hip abductor strength was found to be a risk factor in adolescents but not in adults.^27^ This makes us question the utility of strengthening VM and VL in adolescents with patellofemoral pain, and the lack of strength deficit could explain why an exercise program was found to be less effective in adolescents than adults.^32^ Yet, we currently do not know the mechanisms by which exercise improves patellofemoral pain in adolescents or adults. It may not be by changing specific muscle parameters but rather by other mechanisms (eg, reducing systemic inflammation or psychosocial effects such as reducing kinesiophobia). Taken together, these observations suggest that patellofemoral pain in adolescents may have a different origin than in adults and that the populations may have to be treated differently.

Our force and torque indices provide new and noteworthy knowledge, but their interpretation must be restricted to the context of their measurement. First, our results are valid for the knee position investigated (60° of knee flexion). This angle was chosen because there is no force deficit for adolescents with patellofemoral pain in this position.^33^ Hence, it reduces the possibility of a lower maximal activation in adolescents with patellofemoral pain, which was a requirement for the normalization of EMG.^7^ Yet, patellar maltracking is most evident at lower angles (between 10° and 30° of knee flexion^9^), and it is possible that force distribution is different between groups at lower knee angles and thus not captured in the current study.

Second, our results concern isometric contractions only. During isometric contractions, the force-length relationship of VM and VL, which was not taken into account in this study, also affects the isometric force produced by each muscle. Testing was performed at 60° of knee flexion, where we assumed that both muscles were on the ascendant portion of their force-length relationship, based on VL architectural studies.^20^ Even if there is a lack of similar VM architectural studies, as VM and VL cross the same joint (the knee only), it seemed reasonable to assume that the force imbalance between muscles was minimally affected by a difference in the operating ranges of the muscles over the force-length relationship. During dynamic contractions, muscle force-velocity properties will also affect the force output from individual muscles within a muscle group.^11^ In the absence of experimental techniques to measure the force-velocity relationship of an individual muscle, it is not possible to estimate VM and VL force distribution during dynamic knee extensions. We could hypothesize that a different force distribution is present during dynamic tasks, such as squatting or climbing stairs, for adolescents with patellofemoral pain, as these tasks are known to generate symptoms.

Third, moment arms were measured at rest, and muscle tension produced during contraction could modify the relative VL and VM moment arms—hence the outcomes. However, because of the bony constraints of the trochlea, the patella is stable when the knee is flexed at 60° (our testing angle), and we contend it is unlikely that VL and VM moment arms would have been affected differently between the 2 groups.^42^

## Conclusion

In adolescents with patellofemoral pain, there is no difference in the distribution of VM and VL torque indices compared with matched adolescents with no knee pain during isometric tasks with 60° of knee flexion. Further research on the strength of covariation between VM and VL activation and how this relates to internal patellofemoral joint stress could improve our understanding of the onset and persistence of the condition and provide important insights to optimize future intervention strategies.
